# MicroRNA Seed Region Length Impact on Target Messenger RNA Expression and Survival in Colorectal Cancer

**DOI:** 10.1371/journal.pone.0154177

**Published:** 2016-04-28

**Authors:** Lila E. Mullany, Jennifer S. Herrick, Roger K. Wolff, Martha L. Slattery

**Affiliations:** Department of Medicine, Division of Epidemiology, University of Utah, Salt Lake City, Utah, United States of America; University of São Paulo, BRAZIL

## Abstract

microRNAs (miRNA) repress messenger RNAs post-transcriptionally through binding to the 3’ UTR of the mRNA with the miRNA seed region. It has been purported that longer seed regions have a greater efficacy on mRNA repression. We tested this hypothesis by evaluating differential expression of miRNAs involved in regulating the immune response, an important mechanism in colorectal cancer (CRC), by seed length category. We subsequently evaluated differential expression of these miRNAs’ targets in colonic tissue and the impact of these miRNAs on CRC survival. We determined sequence complementarity between each miRNA seed region and the 3’ UTR of each experimentally verified mRNA target gene. We classified miRNAs into groups based on seed regions matching perfectly to a mRNA UTR with six bases beginning at position two, seven bases beginning at position one, seven bases beginning at position two, or eight bases beginning at position one. We analyzed these groups in terms of miRNA differential expression between carcinoma and normal colorectal mucosa, differential colonic target mRNA expression, and risk of dying from CRC. After correction for multiple comparisons, the proportion of the miRNAs that were associated with differential mRNA expression was 0% for the 6-mer, 13.64% for the 7α-mer group, 12.82% for the 7β-mer group, and 8.70% for the 8-mer group. The proportion of miRNAs associated with survival was 20% for the 6-mer group, 27.27% for the 7α-mer group, 10.23% for the 7β-mer group, and 21.74% for the 8-mer group. We did not see a linear relationship between seed length and miRNA expression dysregulation, mRNA expression, or survival. Our findings do not support the hypothesis the seed region length alone influences mRNA repression.

## Introduction

MicroRNAs (miRNAs) have been increasingly under investigation for the role they play in biological processes, particularly as it pertains to the development and progression of cancers. Mature miRNAs are small (~22–23 nucleotides long), endogenously expressed, non-protein-coding RNA molecules that post-transcriptionally regulate messenger RNAs (mRNAs) [1–3]. They do this by binding to the 3’ untranslated region (UTR) of the mRNA and causing either mRNA degradation, by more precise binding, or inhibition of mRNA translation, by less precise binding [4, 5]. MiRNAs are able to regulate many mRNAs individually [4], and more than one miRNA may target an individual mRNA, creating a complex dynamic of cooperative regulation [6].

In 2015, the American Cancer Society reported that colorectal cancer (CRC) is the second leading cause of cancer-related deaths in the United States when male and females are considered together, and the third when they are considered separately, killing 100,000 people in the U.S. each year [7]. CRC has been shown to be a complex disease, involving many biological processes [8], and as such miRNAs have been postulated as key regulators of CRC [9]. One central process in tumorigenesis that has been proposed to be salient in the development and progression of CRC is chronic inflammation [10], and more broadly, the involvement of the immune response [8, 11]. Tumors are known to be immunogenic, in that they elicit an immune response [11]. In addition to regulating various mRNAs target genes that are involved in the immune response, miRNAs have been implicated in the differentiation of hematopoietic lineages, playing an important role in both B and T cell responses [3].

While many factors may contribute to miRNA-mRNA binding, the completeness with which the miRNA binds to the mRNA, and the resulting impact the miRNA has on mRNA expression, is generally thought to be determined by the seed sequence in the miRNA [1, 12, 13]. The exact definition of the seed region is somewhat debated in the literature but generally the canonical, or “core”, seed region is thought to comprise a contiguous string of at least 6 nucleotides beginning at position two [13]. More specifically, the core seeds have been described as a 6-mer (bases 2–7), 7-mer (“7-mer-A1” being bases 1–7, and “7-mer-m8” being bases 2–8), and 8-mer (bases 1–8); in some reviews the 7-mer-A1 and 8-mer seeds are required to have an adenine, ‘A’, as the first nucleotide [1, 13–16]. Ellwanger et al. found that longer seeds, i.e. seeds of 7 or 8 nucleotides in length, were more evolutionarily conserved than shorter ones [13].

In this study, we look at seed sequence similarity between miRNAs and their corresponding verified mRNA targets that participate in the immune response, and evaluate length of seed matches as it relates to miRNA expression in normal colorectal mucosa, colorectal carcinoma tissue, and differential expression as well miRNA impact on survival and colonic mRNA differential expression. By evaluating a subset of miRNAs, those that target the immune response, we focus our investigation on miRNAs involved in physiological responses important to cancer, and limit the number of interactions we evaluate, for a more targeted approach. As longer seed regions are purported to have a greater efficacy as it pertains to mRNA repression [1], and more precise binding results in mRNA degradation, we hypothesize that miRNAs that bind to target genes with a longer seed region will be more significantly associated with CRC-specific survival and mRNA degradation.

## Materials and Methods

### Study Population

Data come from participants in the population-based Diet, Activity, and Lifestyle study that were recruited from Utah or the Kaiser Permanente Medical Care Program of Northern California (KPMCP). Colon cancer cases were identified as having a primary adenocarcinoma diagnosed between 1 October 1991 and 30 September 1994, while rectal cancer cases, were diagnosed between May 1997 and May 2001. All eligible cases were between 30 and 79 years of age at diagnosis, living in the study area, spoke English, were able to complete an interview, and had no prior history of CRC, Crohn’s disease, ulcerative colitis, or known familial adenomatous polyposis. This study was approved by the Institutional Review Board at the University of Utah; all participants signed an informed consent form.

### miRNA Processing

RNA was extracted from formalin-fixed paraffin embedded tissues and processed as previously described [17]. 100 ng total RNA was labeled with Cy3 and hybridized to Agilent Human miRNA Microarrays V19.0 and were scanned on an Agilent SureScan microarray scanner model G2600D using Agilent Feature Extract software v.11.5.1.1. Data were required to pass stringent QC parameters established by Agilent that included tests for excessive background fluorescence, excessive variation among probe sequence replicates on the array, and measures of the total gene signal on the array to assess low signal. If samples failed to meet QC standards, the sample was repeated, and if a sample failed QC assessment a second time the sample was deemed to be of poor quality and was excluded from down-stream analysis. The Agilent platform was found to be highly reliable (r = 0.98), to have reasonable agreement with NanoString [18] as well as excellent agreement with qRT-PCR (Pellatt, in press). For unpaired samples due to missing normal scans, we imputed values for normal mucosa as previously described in [19]. To minimize differences that could be attributed to the array, amount of RNA, location on array, or other factors that could erroneously influence expression, total gene signal was normalized by multiplying each sample by a scaling factor which was the median of the 75^th^ percentiles of all the samples divided by the 75^th^ percentile of each individual sample [20]. This scaling factor was implemented using SAS 9.4.

### mRNA Sequencing Library Preparation, Sequencing, and Data Processing

RNA library construction was done with the Illumina TruSeq Stranded Total RNA Sample Preparation Kit with Ribo-Zero. The samples were then fragmented and primed for cDNA synthesis, adapters were then ligated onto the cDNA, and the resulting samples were then amplified using PCR; the amplified library was then purified using Agencount AMPure XP beads. A more detailed description of the methods can be found in our previous work [21].

Sequencing was done using an Illumina TruSeq v3 single read flow cell and a 50 cycle single-read sequence run was performed on an Illumina HiSeq instrument. Reads were then aligned to a sequence database containing the human genome (build GRCh37/hg19, February 2009 from genome.ucsc.edu) and alignment was performed using novoalign v2.08.01. Python and a pysam library were used to calculate counts for each exon and UTR of the genes using a list of gene coordinates obtained from http://genome.ucsc.edu. We dropped features that were not expressed in our data or for which the expression was missing for the majority of samples. A more detailed description of the methods can be found in our previous work [21].

### Bioinformatics Analysis

**Fig 1:**
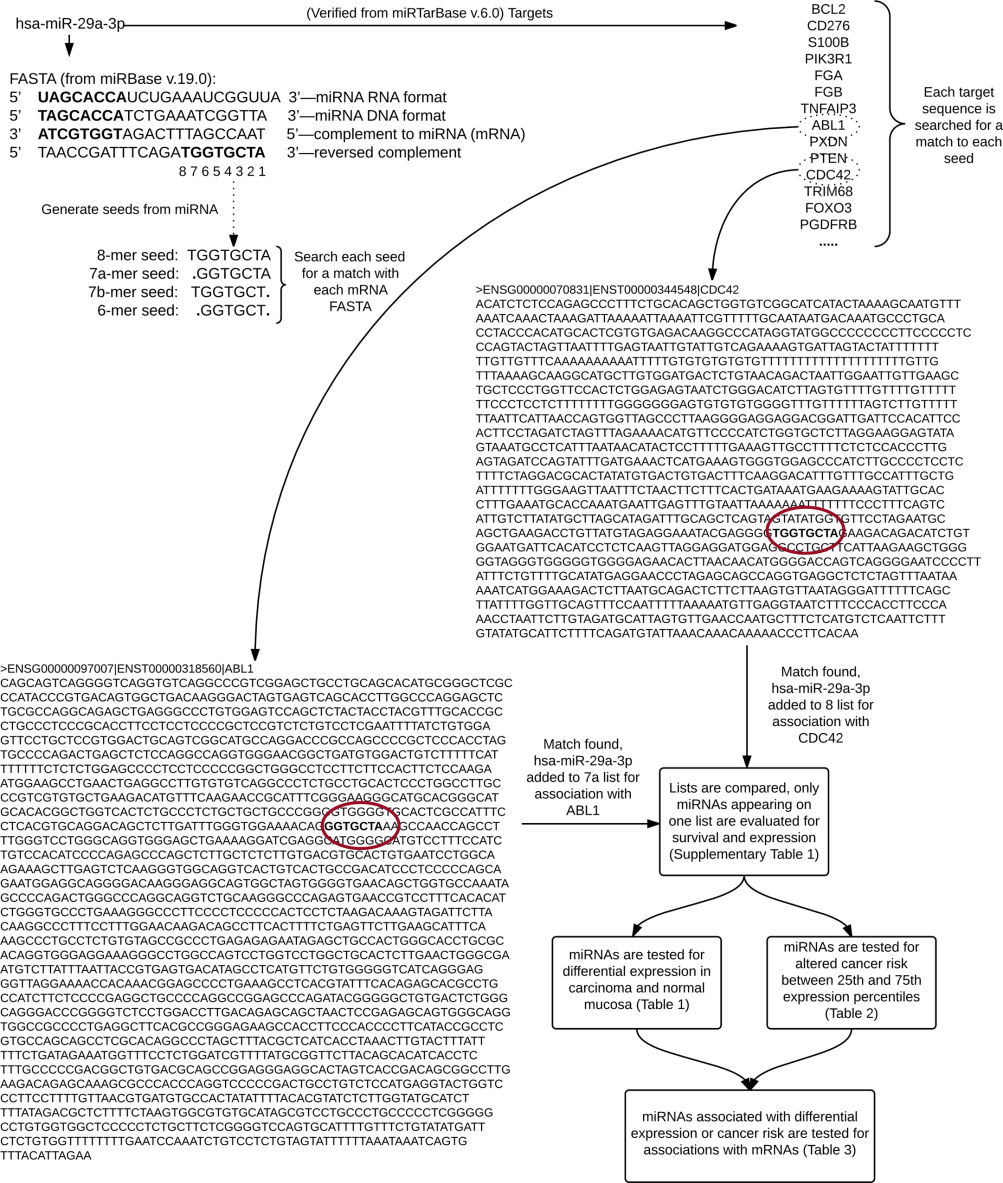
Diagram of miRNA seed generation and matching to mRNA FASTA.

**Fig 2:**
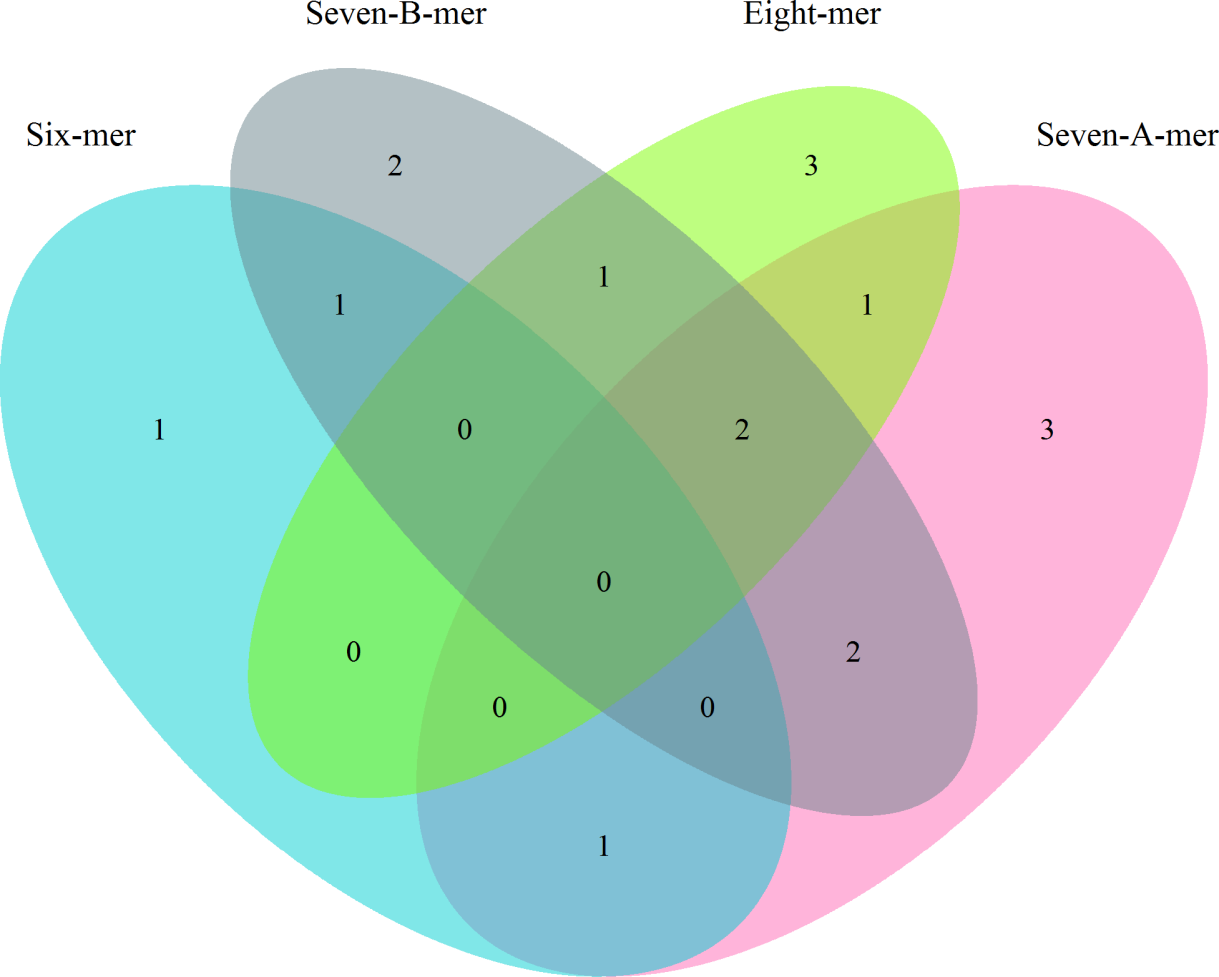
Venn diagram displaying the number of miRNAs in each seed category.

A list of mRNA genes was obtained from Ensembl using Ensembl’s Biomart tool, using the version mapped to human genome GRCh38. We requested a list of Ensembl Gene IDs and Associated Gene Names that had the GO term attribute “immune response”. This yielded a list of 1,762 Ensembl IDs, corresponding to 1,355 unique gene names. A list of miRNA-gene associations was then generated using the Homo sapiens repository from miRTarBase, which utilizes miRBase 21. MiRNAs that were associated with genes found in the ‘immune response’ list and were experimentally verified using either “reporter assay”, “qRT-PCR”, or “western blot”, as these methods are considered stronger by miRTarBase, were further analyzed. These restrictions yielded 1,413 miRNA-target associations comprising 395 unique ‘Immune Response” genes and 327 unique miRNAs. The Agilent platform corresponds to miRBase 19 nomenclature, and by using this version for FASTA sequences we can see which current sequences were being differentially expressed in our dataset. As miRTarBase v6.0 is based off of miRBase 21, this allows us to know the most current associations, and only those miRNAs that matched in name were carried forward in the analysis. MRNA 3’ UTR FASTA sequences were obtained for the genes that were verified targets for miRNAs from Ensembl’s Biomart; we applied a filter requirement of “CCDS ID” in order to obtain consensus sequences only. Seed regions were generated for each miRNA by computing the reverse DNA complement of nucleotides 2–7, 1–7, 2–8, or 1–8 of the 5’ end of the miRNA to make the 6-mer, 7α-mer (also called 7-mer-A1 in the literature), 7β-mer (also called 7-mer-m8 in the literature), and 8-mer seeds respectively. We then searched the 3’ UTR of the mRNA that was experimentally verified to be targeted by the miRNA from which they were generated for each of the seed sequences. MiRNAs that occurred in only one seed category were carried on in the analysis; this was done to polarize the findings as well as reduce ambiguity in inferred miRNA-gene associations. A diagram of this process can be seen in Fig 1. The final miRNA groupings consisted of 99 miRNAs: 15, 22, 39, and 23 miRNAs for 6-mer, 7α-mer, 7β-mer, and 8-mer seeds respectively. The number of miRNAs corresponding to each group can be seen in Fig 2. After determining which miRNAs were significantly differentially expressed between normal colorectal mucosa and colorectal carcinoma tissue, we identified a new set of target genes for those miRNAs. The resulting list was not “immune response”-specific, although it did include “immune response” genes, as the miRNAs in vivo would likely not limit their action on the mRNAs they target. These genes were then analyzed, as described below, for associations with their respective targeting miRNAs to further investigate the impact of seed-type binding on mRNA expression.

### Statistical Methods

Our sample consisted of 1893 subjects with miRNA expression levels for both carcinoma and normal mucosa tissues. We compared log base 2 transformed miRNA expression levels between paired carcinoma tissue and normal colorectal mucosa overall, stratified by study using the significance analysis of microarrays (SAM) technique in the R package siggenes [22]. P-values generated from SAM were based upon 1,000 permutations with a false discovery rate (FDR) set at <0.05 [23]. We analyzed the four seed regions, 6-mer, 7α-mer, 7β-mer, and 8-mer, jointly. For those miRNAs that were significantly differentially expressed, we report the mean level of expression and the fold change (on non-log transformed data) between colorectal carcinoma tissue and normal colorectal mucosa.

Next, we examined the impact of differential miRNA expression on survival using the Cox proportional hazards model in 1855 subjects with survival data available. We calculated hazard ratios (HR) and corresponding 95% confidence intervals (CI) with the unit of change being the interquartile range (IQR), adjusting for age, sex and AJCC stage overall as well as stratified by study and stage. We tested CRC-specific survival based upon months between diagnosis date and death or last follow-up date. Individuals dying of other causes or who were lost to follow-up were censored at their time of death or date of last contact. The R package survival was used to calculate p-values based upon 10,000 permutations of the likelihood ratio test. Because a number of miRNAs were infrequently expressed, which we defined as expression in less than 50% of subjects, we calculated the HR for these miRNA based upon any expression without using permutations to calculate the p-values in SAS 9.4 (SAS Institute, Cary, NC). We combined the p-values of both the more commonly expressed miRNAs and the infrequently expressed miRNAs and adjusted for multiple comparisons using an FDR of 0.05.

Finally, we examined the relationship between differentially expressed miRNAs and their respective mRNA target genes by evaluating the 302 combinations identified in miRTarBase by the method described above in the subset of 148 subjects with mRNA data available. We generated linear regression models adjusted for age and sex on 1,000 bootstrap [24] samples. We calculated the differential mRNA expression as the difference of the log base 2 of the RPKM (Reads per Kilobase per Million) for the carcinoma and normal mucosa tissues. P-values were generated from the distribution of the β coefficients for each miRNA and evaluating H_0_: β = 0 vs. H_1_: β≠0 using the boot package in R. We adjusted for multiple comparisons using an FDR level of 0.05. We standardized the slopes generated from the overall dataset in order to compare the results across the miRNA.

## Results

An overall summary of miRNAs included in the analyses and excluded from analysis after the seed generation can be found in S1 Table.

Forty-three miRNAs were significantly differentially expressed between normal colorectal mucosa and colorectal carcinoma tissues (Table 1). In addition to those differentially expressed for colorectal cancer, three miRNAs were differentially expressed for only colon cancer and three were differentially expressed for only rectal cancer. Of the 49 total dysregulated miRNAs, 18 were upregulated and 31 were downregulated in carcinoma tissue versus normal colorectal mucosa. Six of the miRNAs were classified as 6-mer binding, 12 were classified as 7α-mer, 19 as 7β-mer, and 12 as 8-mers. Sixteen of these miRNAs had a fold change of ≥1.5 or ≤0.67 represented by one 6-mer, four 7α-mers, four 7β-mers, and seven 8-mers.

**Table 1:** miRNAs significantly differentially expressed between carcinoma and normal colonic mucosa in CRC cancer subjects with an FDR <0.05.

	Tumor		Normal		Fold	
miRNA	% Expressing	Mean	% Expressing	Mean	Change(T/N)	Raw p-value
**Six-mer**						
hsa-miR-125b-2-3p	83.6	8.21	87.4	8.81	0.93	<0.0001
hsa-miR-127-3p	17.3	2.33	15.7	0.88	2.66	<0.0001
hsa-miR-192-5p	97.3	84.96	99.4	119.03	0.71	<0.0001
hsa-miR-200b-3p	99.0	145.47	99.4	120.15	1.21	0.008
hsa-miR-486-5p	99.7	16.77	100.0	22.05	0.76	<0.0001
hsa-miR-572	100.0	385.16	100.0	464.38	0.83	<0.0001
**Seven-α-mer**						
hsa-miR-1323	73.4	5.43	75.3	5.79	0.94	0.0014
hsa-miR-197-3p	99.1	14.53	99.6	15.43	0.94	<0.0001
hsa-miR-30e-5p	46.8	2.74	65.7	3.62	0.76	<0.0001
hsa-miR-324-3p	100.0	119.60	100.0	99.82	1.20	<0.0001
hsa-miR-338-3p	3.1	0.95	8.6	1.44	0.66	0.0178
hsa-miR-339-5p	8.8	0.82	6.3	0.15	5.54	<0.0001
hsa-miR-371a-5p	100.0	212.41	100.0	217.98	0.97	0.0108
hsa-miR-425-5p	83.6	9.86	80.3	5.50	1.79	<0.0001
hsa-miR-492	95.4	9.17	98.4	12.49	0.73	<0.0001
hsa-miR-493-3p	69.6	2.60	73.9	2.73	0.95	0.002
hsa-miR-541-3p^R^	2.7	0.12	12.2	0.24	0.51	0.0102
hsa-miR-940	100.0	596.23	100.0	734.71	0.81	<0.0001
**Seven-β-mer**						
hsa-let-7d-5p	97.9	42.97	98.3	30.20	1.42	<0.0001
hsa-miR-125a-3p	100.0	210.57	100.0	233.64	0.90	<0.0001
hsa-miR-126-5p^C^	0.4	0.19	3.4	0.77	0.25	0.0002
hsa-miR-1285-3p	99.6	18.72	99.9	19.61	0.95	0.0003
hsa-miR-135b-5p	23.2	7.21	0.7	0.12	60.96	<0.0001
hsa-miR-150-3p	100.0	237.54	100.0	256.63	0.93	<0.0001
hsa-miR-196a-5p	63.7	6.19	66.9	3.31	1.87	<0.0001
hsa-miR-298	99.9	22.51	100.0	26.52	0.85	<0.0001
hsa-miR-345-5p	100.0	55.05	100.0	61.09	0.90	<0.0001
hsa-miR-361-5p	88.6	10.62	81.6	5.17	2.05	<0.0001
hsa-miR-452-5p	80.0	8.56	84.3	9.69	0.88	<0.0001
hsa-miR-483-5p	100.0	105.07	100.0	95.48	1.10	<0.0001
hsa-miR-490-5p	99.7	18.54	99.8	20.51	0.90	<0.0001
hsa-miR-513a-5p	100.0	391.30	100.0	429.10	0.91	<0.0001
hsa-miR-570-3p^C^	2.5	0.41	14.5	0.57	0.72	<0.0001
hsa-miR-574-3p	100.0	81.12	100.0	82.58	0.98	<0.0001
hsa-miR-638	100.0	3260.48	100.0	3831.57	0.85	<0.0001
hsa-miR-639	99.1	7.41	99.7	8.21	0.90	<0.0001
hsa-miR-99b-5p	47.3	4.83	53.2	3.37	1.43	<0.0001
**Eight-mer**						
hsa-let-7f-5p	98.8	147.33	99.3	106.79	1.38	<0.0001
hsa-miR-142-3p	30.8	2.53	57.5	4.65	0.54	<0.0001
hsa-miR-17-3p	19.5	3.15	0.8	0.12	26.19	<0.0001
hsa-miR-184	34.4	2.64	45.5	2.85	0.93	<0.0001
hsa-miR-186-5p^R^	4.8	1.06	6.5	0.54	1.96	0.0491
hsa-miR-196b-5p	76.8	15.32	71.0	4.72	3.24	<0.0001
hsa-miR-30b-3p	90.7	5.03	87.1	4.52	1.11	<0.0001
hsa-miR-374a-5p	20.9	5.81	4.2	0.53	10.93	<0.0001
hsa-miR-520e	91.4	10.61	97.4	13.60	0.78	<0.0001
hsa-miR-622^R^	100.0	79.01	100.0	85.97	0.92	<0.0001
hsa-miR-645	45.6	3.66	14.9	0.20	17.94	<0.0001
hsa-miR-92a-2-5p^C^	3.6	0.27	16.9	0.48	0.57	<0.0001

^R^Only significant in rectal cancer subjects.

^C^Only significant in colon cancer subjects.

Eighteen miRNAs were significantly associated with altered survival; 17 of these miRNAs were associated with survival after diagnosis with rectal cancer and one was associated with overall CRC survival after adjustment for multiple comparisons (Table 2). Of the 18 miRNAs that were associated with altered survival, 15 also were significantly differentially expressed between normal colorectal mucosa and colorectal carcinoma tissue. No miRNAs altered survival for colon cancer only. Three of these miRNAs were classified as 6-mers, five as 7α-mers, four as 7β-mers, and three as 8-mers.

**Table 2:** miRNAs significantly associated with survival in rectal cancer subjects.

miRNA	% Expressing	25th%ile^1^	75th%ile^1^	HR^2^	95% (CI)		Raw p-value	B-H_adj_ p-value^3^
**6-mer**								
hsa-miR-192-5p	99.4	-1.14	0.18	0.82	(0.72,	0.93)	0.0042	0.0257
hsa-miR-486-5p	100.0	-0.62	-0.17	1.25	(1.08,	1.45)	0.0027	0.0221
hsa-miR-572	100.0	-0.66	-0.10	1.29	(1.12,	1.48)	0.0027	0.0221
**7α-mer**								
hsa-miR-30e-5p	65.7	-1.57	0.42	0.67	(0.55,	0.82)	0.0003	0.0074
hsa-miR-324-3p	100.0	0.06	0.49	1.21	(1.05,	1.40)	0.0078	0.0352
hsa-miR-335-5p	10.1			0.28	(0.12,	0.63)	0.0023	0.0221
hsa-miR-425-5p	80.3	0.00	1.54	0.84	(0.74,	0.95)	0.0094	0.0354
hsa-miR-493-3p	73.9	-0.61	0.76	0.82	(0.70,	0.95)	0.0173	0.0485
hsa-miR-940	100.0	-0.66	-0.06	1.24	(1.06,	1.46)	0.0098	0.0354
**7β-mer**								
hsa-miR-150-3p	100.0	-0.34	0.03	1.22	(1.07,	1.40)	0.0079	0.0352
hsa-miR-361-5p	81.6	0.25	1.55	0.85	(0.76,	0.96)	0.0127	0.0389
hsa-miR-490-5p	99.8	-0.35	0.01	1.16	(1.04,	1.29)	0.0119	0.0389
hsa-miR-638	100.0	-0.60	-0.07	1.26	(1.09,	1.46)	0.0056	0.0305
**8-mer**								
hsa-miR-142-3p	57.5	-1.50	0.00	0.82	(0.71,	0.94)	0.0035	0.0245
hsa-miR-193a-5p	99.8	-0.14	0.20	1.16	(1.04,	1.29)	0.0101	0.0354
hsa-miR-196b-5p	71.0	0.00	3.21	0.72	(0.59,	0.89)	0.0024	0.0221
hsa-miR-374a-5p^o^	4.2			0.46	(0.32,	0.66)	<0.0001	0.0015
hsa-miR-432-5p	54.3	-0.17	0.70	0.89	(0.81,	0.97)	0.0178	0.0485

^o^This miRNA is also significant for overall CRC.

^1^This value is the difference in expression calculated as tumor-normal for both the 25^th^ and 75^th^ percentiles.

^2^We calculated hazard ratios (HR) and corresponding 95% confidence intervals (CI) with the unit of change being the interquartile range (IQR), adjusting for age, sex and AJCC stage overall as well as stratified by study and stage.

^3^Benjamini-Hochberg adjustment for multiple comparisons corrected p-value.

Nineteen miRNAs were associated with expression of their known targeted mRNAs (Table 3); 12 of these remained significant after adjustment for multiple comparisons. Of these 12, six miRNAs were inversely associated with targeted mRNA expression and six were directly associated with targeted mRNA expression. Of the miRNAs that showed a direct association with differential expression of mRNAs, one was classified as a 7α-mer, two as 7β-mers, and three as 8-mers. Of the miRNAs that showed an inverse association with differential mRNA expression two were classified as 7α-mers and four were classified as 7β-mers.

**Table 3:** miRNA-mRNA associations in differential tissue expression between carcinoma tissue and normal colonic mucosa.

miRNA	Target Gene	Beta		Raw P-value	B-H_adj_ p-value^4^^,^^5^	miRNA	Target Gene		Beta	Raw P-value	B-H_adj_ p-value^4^^,^^5^
**hsa-let-7d-5p**	***DICER1***		**0.22**	**0.00**	**0.016**	**hsa-miR-298**	***BACE1***	**-0.27**		**0.00**	**0.006**
**hsa-let-7d-5p**	***HMGA2***		**0.16**	**0.01**	**0.032**	hsa-miR-30e-5p^1^	*BMI1*	0.18		0.03	0.104
**hsa-let-7d-5p**	***SLC11A2***		**0.25**	**<0.0001**	**<0.001**	hsa-miR-30e-5p	*NOTCH1*^*3*^	0.24		0.01	0.080
**hsa-let-7f-5p**	***CCND1***		**0.19**	**0.01**	**0.040**	**hsa-miR-371a-5p**	***CDH1***	**-0.22**		**0.01**	**0.016**
**hsa-let-7f-5p**	***COPS6***		**0.18**	**0.01**	**0.032**	**hsa-miR-371a-5p**	***HSP90AA1***^*3*^	**-0.19**		**0.01**	**0.016**
**hsa-let-7f-5p**	***COPS8***		**0.21**	**<0.0001**	**<0.001**	**hsa-miR-371a-5p**	***PRPF4B***	**-0.16**		**0.03**	**0.045**
hsa-let-7f-5p	*DYRK2*		0.18	0.02	0.069	hsa-miR-425-5p^1^	*CCND1*	0.19		0.03	0.052
**hsa-let-7f-5p**	***ELF4***		**0.24**	**<0.0001**	**<0.001**	**hsa-miR-425-5p**	***FGFR3***^*3*^	**0.20**		**0.01**	**0.024**
**hsa-let-7f-5p**	***MYC***		**0.15**	**0.01**	**0.032**	**hsa-miR-425-5p**	***PTEN***^*3*^	**0.25**		**0.00**	**0.024**
**hsa-let-7f-5p**	***MYH9***		**0.22**	**0.01**	**0.032**	hsa-miR-486-5p^2^	*CD40*	-0.11		0.04	0.114
**hsa-miR-125a-3p**	***GPC4***		**-0.24**	**0.01**	**0.018**	hsa-miR-486-5p	*H3F3B*	-0.14		0.03	0.114
**hsa-miR-1285-3p**	***TP53***^*3*^		**-0.13**	**0.02**	**0.040**	hsa-miR-486-5p	*IGF1R*^*3*^	-0.19		0.01	0.072
hsa-miR-1323	*PTENP1*		-0.17	0.04	0.108	**hsa-miR-492**	***ST6GAL1***^*3*^	**-0.32**		**<0.0001**	**<0.001**
hsa-miR-142-3p^1^	*ARNTL*		0.20	0.00	0.060	hsa-miR-513a-5p	*GNG13*	-0.13		0.04	0.152
hsa-miR-142-3p	*CCNT2*		0.16	0.04	0.270						
**hsa-miR-142-3p**	***KAT2B***		**0.30**	**<0.0001**	**<0.001**						
hsa-miR-142-3p	*TAB2*^*3*^		0.17	0.01	0.080						
**hsa-miR-150-3p**^2^	***IGF1R***^*3*^		**-0.24**	**<0.0001**	**<0.001**						
**hsa-miR-150-3p**	***MYB***		**-0.21**	**<0.0001**	**<0.001**						
hsa-miR-192-5p^1^	*ATP1B1*		0.20	0.02	0.257						
hsa-miR-192-5p	*CUL5*		0.16	0.04	0.257						
hsa-miR-192-5p	*HRH1*		0.14	0.01	0.257						
hsa-miR-192-5p	*RB1*		0.14	0.03	0.257						
hsa-miR-196a-5p	*HMGA1*		0.16	0.04	0.167						
hsa-miR-196a-5p	*HMGA2*		0.15	0.03	0.143						
hsa-miR-196a-5p	*HOXA7*		0.16	0.03	0.143						
**hsa-miR-196a-5p**	***HOXB7***		**0.30**	**<0.0001**	**<0.001**						
**hsa-miR-196a-5p**	***HOXB8***		**0.35**	**<0.0001**	**<0.001**						
**hsa-miR-196b-5p**^1^	***HOXA10***		**0.30**	**<0.0001**	**<0.001**						
**hsa-miR-196b-5p**	***HOXA9***		**0.39**	**<0.0001**	**<0.001**						
hsa-miR-197-3p	*MTHFD1*		-0.13	0.05	0.240						
hsa-miR-200b-3p	*ERBB2IP*		0.20	0.00	0.126						
hsa-miR-200b-3p	*HNRNPA3*		0.15	0.03	0.214						
hsa-miR-200b-3p	*HOXB5*		0.19	0.02	0.214						
hsa-miR-200b-3p	*KDR*		0.17	0.01	0.189						
hsa-miR-200b-3p	*MATR3*		0.16	0.03	0.214						
hsa-miR-200b-3p	*RAB18*		0.16	0.03	0.214						
hsa-miR-200b-3p	*RAB23*		0.17	0.01	0.214						
hsa-miR-200b-3p	*RERE*		0.18	0.03	0.214						
hsa-miR-200b-3p	*RNF2*		0.15	0.04	0.218						
hsa-miR-200b-3p	*ROCK2*		0.17	0.02	0.214						
hsa-miR-200b-3p	*SP1*		0.15	0.03	0.214						

^1^These miRNAs are associated with less risk of dying from cancer (Table 2).

^2^These miRNAs are associated with more risk of dying from cancer (Table 2).

^3^These mRNAs are also ‘immune response’ genes.

^4^Bolded rows are significant at an FDR<0.05.

^5^Benjamini-Hochberg adjustment for multiple comparisons corrected p-value.

A summary of the number of miRNAs significantly differentially expressed, associated with risk of dying from colorectal or rectal cancer, and associated with differential mRNA expression are shown for each seed category in Table 4. While the seed group 7α-mer had the highest proportion of miRNAs in each statistical test, the percentages for each category were very close, suggesting no true difference in proportions. As the total number of miRNAs in each category is relatively small, one or two miRNAs could explain the difference in proportions.

**Table 4:** Proportions of miRNAs from each seed category that were differentially expressed between carcinoma tissue and normal colonic mucosa, were associated with risk of dying from CRC, or were associated with differential mRNA expression.

	6-mer	7α-mer	7β-mer	8-mer
**Total Unique miRNAs in Group**	**15**	**22**	**39**	**23**
**# Sig. Differentially**^**1**^ **Expressed**	6	12	19	12
**% of Total miRNAs in Group**	40.00	54.55	48.72	52.17
**# Associated with Survival**^**2**^	3	6	4	5
**% of Total miRNAs in Group**	20.00	27.27	10.26	21.74
**# Associated with mRNA Expression**^**3**^	3	6	7	3
**% of Total miRNAs in Group**	20.00	27.27	17.95	13.04
**# Associated with mRNA Expression**^**4**^	0	3	5	2
**% of Total miRNAs in Group**	0.00	13.64	12.82	8.70
**Inversely Correlated with mRNA**^**3**^	1	4	5	0
**% of Total miRNAs in Group**	6.67	18.18	12.82	0.00
**Directly Correlated with mRNA**^**3**^	2	2	2	3
**% of Total miRNAs in Group**	13.33	9.09	5.13	13.04
**Inversely Correlated with mRNA**^**4**^	0	2	4	0
**% of Total miRNAs in Group**	0.00	9.09	10.26	0.00
**Directly Correlated with mRNA**^**4**^	0	1	2	3
**% of Total miRNAs in Group**	0.00	4.55	5.13	13.04

^1^This total includes all unique miRNAs found to be differentially expressed in colorectal (overall), colon, or rectal tissues.

^2^This total includes all unique miRNAs found to be associated with survival in colorectal (overall), colon, or rectal tissues.

^3^Including those not significant after multiple comparisons

^4^Only including those significant after multiple comparisons

**Fig 3:**
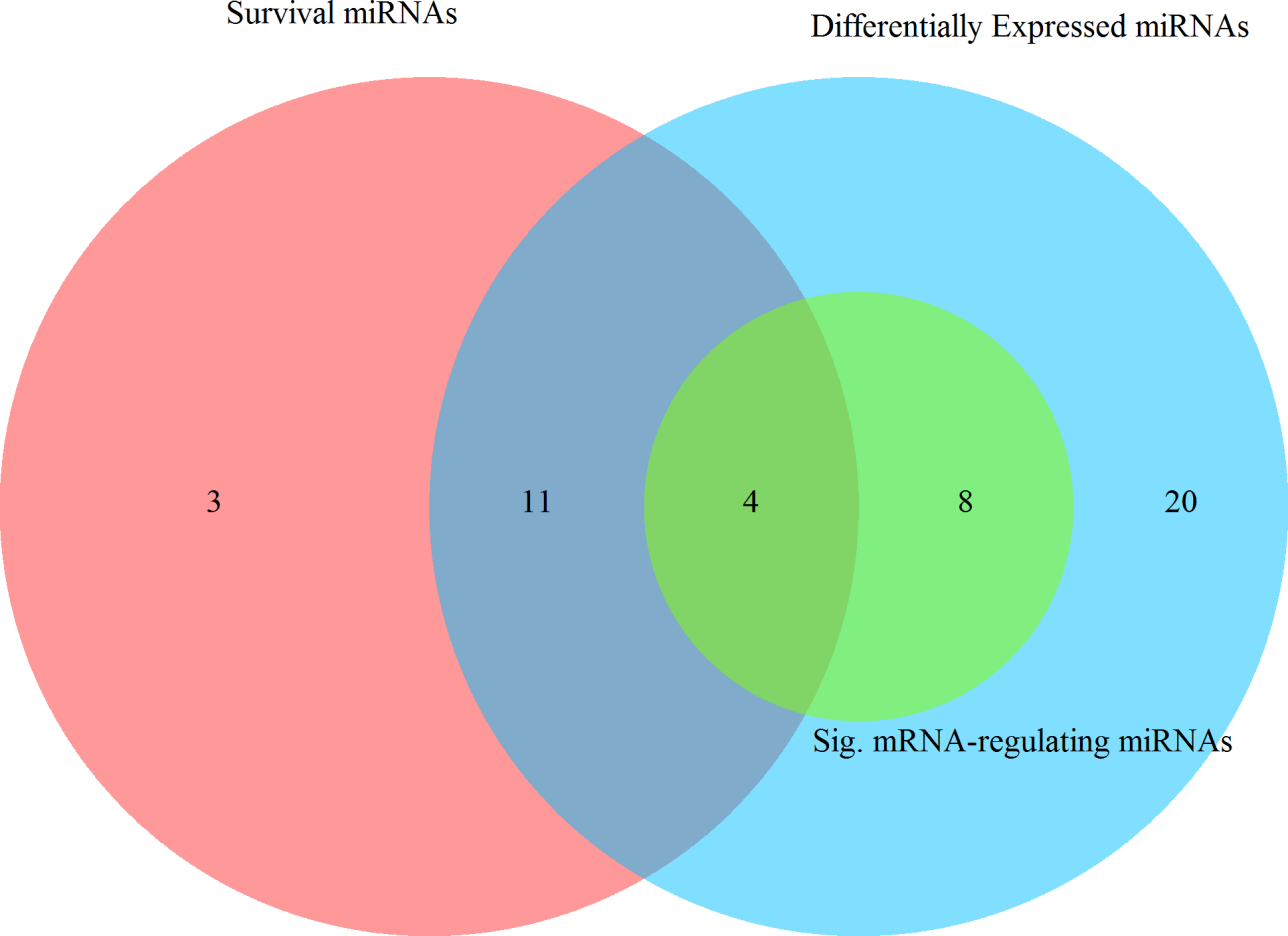
Venn diagram displaying the number of mRNA-regulating miRNAs, miRNAs associated with survival, and miRNAs significantly differentially expressed between carcinoma and normal colonic mucosa.

The overlap of miRNAs represented by each of the analyses can be seen in Fig 3. From this diagram, we see that only four miRNAs were significantly differentially expressed, were associated with altered survival, and were significantly associated with altered mRNA expression after adjustment for multiple comparisons. Of these four miRNAs, two were classified as 8-mers, one as 7β-mer, and one was classified as 7α-mer. Only one of these four, hsa-miR-150-3p, was inversely associated with mRNA expression, the rest were directly associated.

## Discussion

Many miRNAs in this study were differentially expressed between colorectal carcinoma tissue and normal colorectal mucosa, were associated with altered risk of dying from either CRC or rectal cancer, or were associated with differential colonic mRNA expression. Four of these miRNAs were found to have significant associations in all three tests. We hypothesized that, because longer seed regions have a greater efficacy of mRNA repression and more precise binding results in mRNA degradation, that the longer seed regions would be associated more with mRNA differential expression as well as CRC risk. In general, the seed group 7α-mer, which consists of miRNAs with seeds comprising nucleotides 1–7, had more significant findings in terms of how many miRNAs belonging to each category were associated with either miRNA differential expression, mRNA differential expression or survival. However, as the percentages are fairly close for all groups, it is likely that this finding is not statistically different. Additionally, there was no linear trend, i.e. number from 6-seed < number from 7α/β-seeds < number from 8-seeds or vice versa, to the proportion of miRNAs from each seed group that were significant with mRNA differential expression or with survival. This suggests that seed length alone, at least as it is defined in this study, is not associated with either mRNA differential expression between colon carcinoma tissue and normal colonic mucosa or with risk of dying from CRC. There was a slight linear trend in miRNA differential expression with fold changes ≥1.5 or ≤0.67 as it pertains to seed region length, with successively more miRNAs being differentially expressed as seed region length increased. This, however, is unlikely to be biologically relevant.

Contrary to their well-established role as repressors, miRNAs have been recently shown to activate mRNA translation when cells are in a quiescent state [25]. Specifically, exogenous hsa-let-7 was shown to increase *HMGA2* translation when added to serum-starved (quiescent) HeLa cells. In our study we observed increased expression of hsa-let-7d-5p in colorectal carcinoma tissue as compared to normal colorectal mucosa, and a direct association between hsa-let-7f-5p and *HGMA2* expression (β = 0.16, P_adj_ = 0.032), indicating a subsequent increase in *HGMA2* expression in carcinoma tissue. Hsa-let-7d-5p is categorized as a 7β-mer binding miRNA in our dataset. As this dynamic was previously seen in quiescent cells, it may be that subjects who express higher levels of hsa-let-7d-5p and *HMGA2* have more cells that are in a dormant state. We saw upregulation of hsa-miR-196a-5p and hsa-miR-196b-5p in colorectal carcinoma tissue as compared to normal colorectal mucosa; we subsequently saw a direct relationship with hsa-miR-196a-5p and *HOXB7* and hsa-miR-196b-5p with *HOXA10* in colonic carcinoma tissue. The same relationships between these miRNAs and mRNAs were observed in patients with Huntington’s disease as compared to normal brains [26]; this study attributed this observed dynamic to aberrant mRNA processing. As this study identifies a key pathway enriched by these genes as “Cell death and Survival”, found using IPA, it could be that this unorthodox relationship is due to alterations in this pathway. Phosphatase and tensin homolog, or *PTEN*, encodes for an enzyme that is found in many of the body’s tissues and acts to inhibit uncontrolled growth, thus acting as a tumor suppressor [27]. This gene was directly associated with hsa-miR-425-5p, which was increased in carcinoma tissue (fold change = 1.79, P-value < 0.0001). Over expression of hsa-miR-425-5p in colorectal carcinoma was associated with decreased risk of dying from rectal cancer (IQR Range HR 0.84 95% CI 0.74, 0.95 P_adj_ = 0.0354). As this miRNA was associated with increased expression of *PTEN* in colon tissue (β = 0.25, P_adj_ = 0.024), and differential expression of this miRNA was similar for colon and rectal tissue, it is possible that a similar association with PTEN would be seen in rectal tissue. The altered risk of rectal cancer could therefore be impacted by PTEN’s ability to suppress uncontrolled growth.

A limitation is that we evaluated mRNA data for colon tissue only. While we have shown that dysregulation of miRNA is similar for colon and rectal cancer [18], it is unknown if the dysregulation of mRNA is the same for rectal and colon cancer. However, if it is, it is interesting to note that, of the miRNAs that were associated with both altered survival after diagnosis with rectal cancer and mRNA expression in colon tissue, those that increased survival (hsa-miR-192-5p, hsa-miR-30e-5p, hsa-miR-425-5p, hsa-miR-142-3p, and hsa-miR-196b-5p) had direct associations with mRNA expression (β>0) and those that increased risk of death from cancer (hsa-miR-486-5p and hsa-miR-150-3p) had an inverse association with mRNA expression (β<0). As a quiescent cell is not dividing uncontrollably, it is possible that this relationship between miRNAs and mRNA activation in quiescent cells contributes to the improved survival in these subjects, however as some of these genes have oncogenic properties (*MYC*, *MYB*, and *IGF1R*) or are related to DNA transcription and cellular differentiation (*KAT2B*, *HOXB7*, *HOXB8*, *HOXA7*, *HOXA9*, and *HOXA10*) it is likely that many factors influence the overall gene expression in the cell and the subsequent impact on survival. Also, because all of the miRNAs associated with altered risk and mRNA expression are from different seed groups, it is unlikely that seed binding alone contributes to this occurrence.

This study evaluates only CRC tissue. While we believe that the seed regions should behave similarly in other tissues, miRNA expression has been shown to be tissue specific and this may limit the generalization of these findings as applied to other tissue types. There are many possible factors that may also influence miRNA-mRNA binding. As we were interested in how seed region length impacted miRNA binding, mRNA expression, and survival in CRC patients, there are some potentially relevant elements that we did not consider in this study. These influences on miRNA-mRNA binding include: other microRNA-recognition elements (MREs), AU composition and AU-rich elements (AREs) and other influences on site accessibility, 3’ (of the miRNA) compensatory binding, GC content of the seed region, whether the seed sequence is conserved, and distance from the stop codon in and location near the end of the 3’ UTR of the mRNA [1, 2, 15, 28]. Additionally, it may be that some miRNAs primarily interact with mRNAs through their 3’ end, or exhibit non-canonical binding sites (which are sometimes in the literature depicted as seeds that are 6 nucleotides long instead of 7 or 8), manifested by bulges or single-nucleotide loops in the seed regions, or bind in the middle region of the miRNA [16]. These variations on miRNA-mRNA binding were not investigated in this study, and as such some interactions may be missed. Finally, as previously stated, the 7α-mer (described elsewhere as 7-mer-A1) and 8-mer seeds are in some studies depicted as those that have an adenine in position one. We did not calculate seed regions in this manner, instead we found exact, Watson-Crick, matches between the miRNA seeds and mRNAs. Because this distinction is typically applied to mRNA target prediction, and we only looked for seed matches in mRNAs that were experimentally verified as targets for miRNAs that were differentially expressed, we do not feel that this is a detriment to our investigation. Some of the miRNAs, those that begin with a uracil, have complementary sequences beginning with an adenine, and as such our dataset includes some 7α-mers and 8-mers that do follow this standard, and others that follow the broader Watson-Crick pairing. The approach we took in examining seed region similarities looked at perfect matching and intentionally polarized the dataset by only analyzing miRNAs appearing in one seed category. While this was done in order to limit comparisons and make interpretation of the results easier, it could be that this approach is not appropriate for the desired analysis. It is possible that an approach that determines a consensus sequence or sequences and allows for miRNAs to be in multiple groups would be better and we encourage other studies to examine this.

## Conclusions

We hypothesized that the miRNAs belonging to the longer seed groups, i.e. those in the 7 and 8-seed categories, would comprise the majority of the findings for mRNA differential expression as well as for survival, due to their proposed capacity for mRNA repression. While many miRNAs were differentially expressed between carcinoma tissue and normal colorectal mucosa, were associated with risk of dying after a diagnosis with rectal cancer, or were associated with differential mRNA expression, seed region length did not influence these associations. Our findings could be influenced by the manner in which we determined seed region and which miRNAs to include in the analysis. We found that miRNAs that were directly associated with colonic mRNA expression increased survival from rectal cancer, and those that were inversely associated with colonic mRNA expression increased risk of dying from CRC. Because of the small number of miRNAs that were associated with CRC survival, we encourage other studies to investigate this dynamic.

## Supporting Information

S1 Table Summary of miRNA outcomes and seed region group. (DOCX)
