# Supplementary material for: MicroRNA Seed Region Length Impact on Target Messenger RNA Expression and Survival in Colorectal Cancer
Source: PLoS One. 2016 Apr 28;11(4):e0154177. doi: 10.1371/journal.pone.0154177 (PMC4849741; doi:10.1371/journal.pone.0154177)
Supplement: S1 Table — (DOCX) [file pone.0154177.s001.docx]

| **miRNA** | **Seed Group** | **Differentially Expressed** | **Associated with Survival** | **Associated with mRNA Expression^1^** |
| --- | --- | --- | --- | --- |
| hsa-miR-125b-2-3p | 6 | x |  |  |
| hsa-miR-127-3p | 6 | x |  |  |
| hsa-miR-142-5p | 6 |  |  |  |
| hsa-miR-192-5p | 6 | x | x | x |
| hsa-miR-19b-1-5p | 6 |  |  |  |
| hsa-miR-200b-3p | 6 | x |  | x |
| hsa-miR-24-2-5p | 6 |  |  |  |
| hsa-miR-363-5p | 6 |  |  |  |
| hsa-miR-380-5p | 6 |  |  |  |
| hsa-miR-421 | 6 |  |  |  |
| hsa-miR-486-5p | 6 | x | x | x |
| hsa-miR-543 | 6 |  |  |  |
| hsa-miR-572 | 6 | x | x |  |
| hsa-miR-657 | 6 |  |  |  |
| hsa-miR-767-5p | 6 |  |  |  |
| hsa-miR-1323 | 7a | x |  | x |
| hsa-miR-146b-3p | 7a |  |  |  |
| hsa-miR-155-3p | 7a |  |  |  |
| hsa-miR-197-3p | 7a | x |  | x |
| hsa-miR-27a-5p | 7a |  |  |  |
| hsa-miR-30a-3p | 7a |  |  |  |
| hsa-miR-30e-3p | 7a | x | x |  |
| hsa-miR-30e-5p | 7a | x |  | x |
| hsa-miR-324-3p | 7a | x | x |  |
| hsa-miR-335-5p | 7a |  | x |  |
| hsa-miR-338-3p | 7a | x |  |  |
| hsa-miR-339-5p | 7a | x |  |  |
| hsa-miR-371a-5p | 7a | x |  | x |
| hsa-miR-382-5p | 7a |  |  |  |
| hsa-miR-425-5p | 7a | x | x | x |
| hsa-miR-492 | 7a | x |  | x |
| hsa-miR-493-3p | 7a | x | x |  |
| hsa-miR-519e-3p | 7a |  |  |  |
| hsa-miR-520a-3p | 7a |  |  |  |
| hsa-miR-541-3p | 7a | x |  |  |
| hsa-miR-612 | 7a |  |  |  |
| hsa-miR-940 | 7a | x | x |  |
| hsa-let-7d-5p | 7b | x |  | x |
| hsa-miR-101-5p | 7b |  |  |  |
| hsa-miR-125a-3p | 7b | x |  | x |
| hsa-miR-126-5p | 7b | x |  |  |
| hsa-miR-1285-3p | 7b | x |  | x |
| hsa-miR-135b-5p | 7b | x |  |  |
| hsa-miR-138-5p | 7b |  |  |  |
| hsa-miR-150-3p | 7b | x | x | x |
| hsa-miR-154-5p | 7b |  |  |  |
| hsa-miR-191-5p | 7b |  |  |  |
| hsa-miR-196a-5p | 7b | x |  | x |
| hsa-miR-212-3p | 7b |  |  |  |
| hsa-miR-296-3p | 7b |  |  |  |
| hsa-miR-298 | 7b | x |  | x |
| hsa-miR-299-5p | 7b |  |  |  |
| hsa-miR-33a-5p | 7b |  |  |  |
| hsa-miR-345-5p | 7b | x |  |  |
| hsa-miR-361-5p | 7b | x | x |  |
| hsa-miR-362-3p | 7b |  |  |  |
| hsa-miR-452-5p | 7b | x |  |  |
| hsa-miR-483-5p | 7b | x |  |  |
| hsa-miR-485-5p | 7b |  |  |  |
| hsa-miR-490-5p | 7b | x | x |  |
| hsa-miR-502-5p | 7b |  |  |  |
| hsa-miR-513a-5p | 7b | x |  | x |
| hsa-miR-515-3p | 7b |  |  |  |
| hsa-miR-517a-3p | 7b |  |  |  |
| hsa-miR-517c-3p | 7b |  |  |  |
| hsa-miR-524-5p | 7b |  |  |  |
| hsa-miR-539-5p | 7b |  |  |  |
| hsa-miR-559 | 7b |  |  |  |
| hsa-miR-570-3p | 7b | x |  |  |
| hsa-miR-574-3p | 7b | x |  |  |
| hsa-miR-591 | 7b |  |  |  |
| hsa-miR-638 | 7b | x | x |  |
| hsa-miR-639 | 7b | x |  |  |
| hsa-miR-654-3p | 7b |  |  |  |
| hsa-miR-941 | 7b |  |  |  |
| hsa-miR-99b-5p | 7b | x |  |  |
| hsa-let-7f-5p | 8 | x |  | x |
| hsa-miR-142-3p | 8 | x | x | x |
| hsa-miR-17-3p | 8 | x |  |  |
| hsa-miR-184 | 8 | x |  |  |
| hsa-miR-186-5p | 8 | x |  |  |
| hsa-miR-18a-3p | 8 |  |  |  |
| hsa-miR-193a-5p | 8 |  | x |  |
| hsa-miR-196b-5p | 8 | x | x | x |
| hsa-miR-301a-3p | 8 |  |  |  |
| hsa-miR-30b-3p | 8 | x |  |  |
| hsa-miR-323b-5p | 8 |  |  |  |
| hsa-miR-340-5p | 8 |  |  |  |
| hsa-miR-374a-5p | 8 | x | x |  |
| hsa-miR-377-3p | 8 |  |  |  |
| hsa-miR-432-5p | 8 |  | x |  |
| hsa-miR-4723-5p | 8 |  |  |  |
| hsa-miR-495-3p | 8 |  |  |  |
| hsa-miR-519b-3p | 8 |  |  |  |
| hsa-miR-520e | 8 | x |  |  |
| hsa-miR-582-5p | 8 |  |  |  |
| hsa-miR-622 | 8 | x |  |  |
| hsa-miR-645 | 8 | x |  |  |
| hsa-miR-92a-2-5p | 8 | x |  |  |
| hsa-miR-129-5p | 6, 7a | N/A^2^ | N/A | N/A |
| hsa-miR-133b | 6, 7a | N/A | N/A | N/A |
| hsa-miR-1915-3p | 6, 7a | N/A | N/A | N/A |
| hsa-miR-193a-3p | 6, 7a | N/A | N/A | N/A |
| hsa-miR-363-3p | 6, 7a | N/A | N/A | N/A |
| hsa-miR-548d-3p | 6, 7a | N/A | N/A | N/A |
| hsa-miR-665 | 6, 7a | N/A | N/A | N/A |
| hsa-miR-183-5p | 6, 7b | N/A | N/A | N/A |
| hsa-miR-18b-5p | 6, 7b | N/A | N/A | N/A |
| hsa-miR-302a-3p | 6, 7b | N/A | N/A | N/A |
| hsa-miR-331-3p | 6, 7b | N/A | N/A | N/A |
| hsa-miR-376a-3p | 6, 7b | N/A | N/A | N/A |
| hsa-miR-608 | 6, 7b | N/A | N/A | N/A |
| hsa-miR-200c-3p | 6, 8 | N/A | N/A | N/A |
| hsa-miR-26b-5p | 6, 8 | N/A | N/A | N/A |
| hsa-miR-376c-3p | 6, 8 | N/A | N/A | N/A |
| hsa-miR-644a | 6, 8 | N/A | N/A | N/A |
| hsa-miR-92a-3p | 6, 8 | N/A | N/A | N/A |
| hsa-miR-92b-3p | 6, 8 | N/A | N/A | N/A |
| hsa-miR-216a-5p | 7a, 7b | N/A | N/A | N/A |
| hsa-miR-22-3p | 7a, 7b | N/A | N/A | N/A |
| hsa-miR-302d-3p | 7a, 7b | N/A | N/A | N/A |
| hsa-miR-326 | 7a, 7b | N/A | N/A | N/A |
| hsa-miR-330-3p | 7a, 7b | N/A | N/A | N/A |
| hsa-miR-499a-5p | 7a, 7b | N/A | N/A | N/A |
| hsa-miR-520h | 7a, 7b | N/A | N/A | N/A |
| hsa-miR-630 | 7a, 7b | N/A | N/A | N/A |
| hsa-miR-663a | 7a, 7b | N/A | N/A | N/A |
| hsa-let-7b-5p | 7a, 8 | N/A | N/A | N/A |
| hsa-let-7e-5p | 7a, 8 | N/A | N/A | N/A |
| hsa-miR-105-5p | 7a, 8 | N/A | N/A | N/A |
| hsa-miR-194-5p | 7a, 8 | N/A | N/A | N/A |
| hsa-miR-28-5p | 7a, 8 | N/A | N/A | N/A |
| hsa-miR-30a-5p | 7a, 8 | N/A | N/A | N/A |
| hsa-miR-30c-5p | 7a, 8 | N/A | N/A | N/A |
| hsa-miR-30d-5p | 7a, 8 | N/A | N/A | N/A |
| hsa-miR-448 | 7a, 8 | N/A | N/A | N/A |
| hsa-miR-451a | 7a, 8 | N/A | N/A | N/A |
| hsa-miR-491-5p | 7a, 8 | N/A | N/A | N/A |
| hsa-miR-519a-3p | 7a, 8 | N/A | N/A | N/A |
| hsa-miR-519c-3p | 7a, 8 | N/A | N/A | N/A |
| hsa-miR-520b | 7a, 8 | N/A | N/A | N/A |
| hsa-miR-758-3p | 7a, 8 | N/A | N/A | N/A |
| hsa-miR-98-5p | 7a, 8 | N/A | N/A | N/A |
| hsa-miR-100-5p | 7b, 8 | N/A | N/A | N/A |
| hsa-miR-140-5p | 7b, 8 | N/A | N/A | N/A |
| hsa-miR-148b-3p | 7b, 8 | N/A | N/A | N/A |
| hsa-miR-149-3p | 7b, 8 | N/A | N/A | N/A |
| hsa-miR-149-5p | 7b, 8 | N/A | N/A | N/A |
| hsa-miR-187-3p | 7b, 8 | N/A | N/A | N/A |
| hsa-miR-19a-3p | 7b, 8 | N/A | N/A | N/A |
| hsa-miR-20b-5p | 7b, 8 | N/A | N/A | N/A |
| hsa-miR-24-3p | 7b, 8 | N/A | N/A | N/A |
| hsa-miR-302c-3p | 7b, 8 | N/A | N/A | N/A |
| hsa-miR-32-5p | 7b, 8 | N/A | N/A | N/A |
| hsa-miR-34b-3p | 7b, 8 | N/A | N/A | N/A |
| hsa-miR-373-3p | 7b, 8 | N/A | N/A | N/A |
| hsa-miR-520c-3p | 7b, 8 | N/A | N/A | N/A |
| hsa-miR-661 | 7b, 8 | N/A | N/A | N/A |
| hsa-miR-99a-5p | 7b, 8 | N/A | N/A | N/A |
| hsa-miR-106b-5p | 6, 7a, 7b | N/A | N/A | N/A |
| hsa-miR-126-3p | 6, 7a, 7b | N/A | N/A | N/A |
| hsa-miR-148a-3p | 6, 7a, 7b | N/A | N/A | N/A |
| hsa-miR-18a-5p | 6, 7a, 7b | N/A | N/A | N/A |
| hsa-miR-199a-3p | 6, 7a, 7b | N/A | N/A | N/A |
| hsa-miR-199b-5p | 6, 7a, 7b | N/A | N/A | N/A |
| hsa-miR-206 | 6, 7a, 7b | N/A | N/A | N/A |
| hsa-miR-222-3p | 6, 7a, 7b | N/A | N/A | N/A |
| hsa-miR-33b-5p | 6, 7a, 7b | N/A | N/A | N/A |
| hsa-miR-34b-5p | 6, 7a, 7b | N/A | N/A | N/A |
| hsa-miR-362-5p | 6, 7a, 7b | N/A | N/A | N/A |
| hsa-miR-503-5p | 6, 7a, 7b | N/A | N/A | N/A |
| hsa-miR-124-3p | 6, 7a, 8 | N/A | N/A | N/A |
| hsa-miR-137 | 6, 7a, 8 | N/A | N/A | N/A |
| hsa-miR-144-3p | 6, 7a, 8 | N/A | N/A | N/A |
| hsa-miR-146b-5p | 6, 7a, 8 | N/A | N/A | N/A |
| hsa-miR-193b-3p | 6, 7a, 8 | N/A | N/A | N/A |
| hsa-miR-30b-5p | 6, 7a, 8 | N/A | N/A | N/A |
| hsa-miR-320a | 6, 7a, 8 | N/A | N/A | N/A |
| hsa-miR-375 | 6, 7a, 8 | N/A | N/A | N/A |
| hsa-miR-10b-5p | 6, 7b, 8 | N/A | N/A | N/A |
| hsa-miR-130a-3p | 6, 7b, 8 | N/A | N/A | N/A |
| hsa-miR-130b-3p | 6, 7b, 8 | N/A | N/A | N/A |
| hsa-miR-136-5p | 6, 7b, 8 | N/A | N/A | N/A |
| hsa-miR-15b-5p | 6, 7b, 8 | N/A | N/A | N/A |
| hsa-miR-195-5p | 6, 7b, 8 | N/A | N/A | N/A |
| hsa-miR-19b-3p | 6, 7b, 8 | N/A | N/A | N/A |
| hsa-miR-23a-3p | 6, 7b, 8 | N/A | N/A | N/A |
| hsa-miR-25-3p | 6, 7b, 8 | N/A | N/A | N/A |
| hsa-miR-34c-5p | 6, 7b, 8 | N/A | N/A | N/A |
| hsa-miR-708-5p | 6, 7b, 8 | N/A | N/A | N/A |
| hsa-let-7i-5p | 7a, 7b, 8 | N/A | N/A | N/A |
| hsa-miR-101-3p | 7a, 7b, 8 | N/A | N/A | N/A |
| hsa-miR-103a-3p | 7a, 7b, 8 | N/A | N/A | N/A |
| hsa-miR-132-3p | 7a, 7b, 8 | N/A | N/A | N/A |
| hsa-miR-135a-5p | 7a, 7b, 8 | N/A | N/A | N/A |
| hsa-miR-139-5p | 7a, 7b, 8 | N/A | N/A | N/A |
| hsa-miR-150-5p | 7a, 7b, 8 | N/A | N/A | N/A |
| hsa-miR-185-5p | 7a, 7b, 8 | N/A | N/A | N/A |
| hsa-miR-204-5p | 7a, 7b, 8 | N/A | N/A | N/A |
| hsa-miR-217 | 7a, 7b, 8 | N/A | N/A | N/A |
| hsa-miR-218-5p | 7a, 7b, 8 | N/A | N/A | N/A |
| hsa-miR-223-3p | 7a, 7b, 8 | N/A | N/A | N/A |
| hsa-miR-26a-5p | 7a, 7b, 8 | N/A | N/A | N/A |
| hsa-miR-27a-3p | 7a, 7b, 8 | N/A | N/A | N/A |
| hsa-miR-296-5p | 7a, 7b, 8 | N/A | N/A | N/A |
| hsa-miR-29a-3p | 7a, 7b, 8 | N/A | N/A | N/A |
| hsa-miR-29b-3p | 7a, 7b, 8 | N/A | N/A | N/A |
| hsa-miR-302b-3p | 7a, 7b, 8 | N/A | N/A | N/A |
| hsa-miR-31-5p | 7a, 7b, 8 | N/A | N/A | N/A |
| hsa-miR-378a-3p | 7a, 7b, 8 | N/A | N/A | N/A |
| hsa-miR-424-5p | 7a, 7b, 8 | N/A | N/A | N/A |
| hsa-miR-449a | 7a, 7b, 8 | N/A | N/A | N/A |
| hsa-miR-497-5p | 7a, 7b, 8 | N/A | N/A | N/A |
| hsa-miR-500a-5p | 7a, 7b, 8 | N/A | N/A | N/A |
| hsa-miR-93-5p | 7a, 7b, 8 | N/A | N/A | N/A |
| hsa-let-7a-5p | 6, 7a, 7b, 8 | N/A | N/A | N/A |
| hsa-let-7g-5p | 6, 7a, 7b, 8 | N/A | N/A | N/A |
| hsa-miR-106a-5p | 6, 7a, 7b, 8 | N/A | N/A | N/A |
| hsa-miR-107 | 6, 7a, 7b, 8 | N/A | N/A | N/A |
| hsa-miR-10a-5p | 6, 7a, 7b, 8 | N/A | N/A | N/A |
| hsa-miR-122-5p | 6, 7a, 7b, 8 | N/A | N/A | N/A |
| hsa-miR-125a-5p | 6, 7a, 7b, 8 | N/A | N/A | N/A |
| hsa-miR-125b-5p | 6, 7a, 7b, 8 | N/A | N/A | N/A |
| hsa-miR-141-3p | 6, 7a, 7b, 8 | N/A | N/A | N/A |
| hsa-miR-143-3p | 6, 7a, 7b, 8 | N/A | N/A | N/A |
| hsa-miR-145-5p | 6, 7a, 7b, 8 | N/A | N/A | N/A |
| hsa-miR-146a-5p | 6, 7a, 7b, 8 | N/A | N/A | N/A |
| hsa-miR-155-5p | 6, 7a, 7b, 8 | N/A | N/A | N/A |
| hsa-miR-15a-5p | 6, 7a, 7b, 8 | N/A | N/A | N/A |
| hsa-miR-16-5p | 6, 7a, 7b, 8 | N/A | N/A | N/A |
| hsa-miR-17-5p | 6, 7a, 7b, 8 | N/A | N/A | N/A |
| hsa-miR-181a-5p | 6, 7a, 7b, 8 | N/A | N/A | N/A |
| hsa-miR-181b-5p | 6, 7a, 7b, 8 | N/A | N/A | N/A |
| hsa-miR-181c-5p | 6, 7a, 7b, 8 | N/A | N/A | N/A |
| hsa-miR-182-5p | 6, 7a, 7b, 8 | N/A | N/A | N/A |
| hsa-miR-199a-5p | 6, 7a, 7b, 8 | N/A | N/A | N/A |
| hsa-miR-200a-3p | 6, 7a, 7b, 8 | N/A | N/A | N/A |
| hsa-miR-205-5p | 6, 7a, 7b, 8 | N/A | N/A | N/A |
| hsa-miR-20a-5p | 6, 7a, 7b, 8 | N/A | N/A | N/A |
| hsa-miR-211-5p | 6, 7a, 7b, 8 | N/A | N/A | N/A |
| hsa-miR-214-3p | 6, 7a, 7b, 8 | N/A | N/A | N/A |
| hsa-miR-21-5p | 6, 7a, 7b, 8 | N/A | N/A | N/A |
| hsa-miR-221-3p | 6, 7a, 7b, 8 | N/A | N/A | N/A |
| hsa-miR-224-5p | 6, 7a, 7b, 8 | N/A | N/A | N/A |
| hsa-miR-23b-3p | 6, 7a, 7b, 8 | N/A | N/A | N/A |
| hsa-miR-27b-3p | 6, 7a, 7b, 8 | N/A | N/A | N/A |
| hsa-miR-29c-3p | 6, 7a, 7b, 8 | N/A | N/A | N/A |
| hsa-miR-34a-5p | 6, 7a, 7b, 8 | N/A | N/A | N/A |
| hsa-miR-365a-3p | 6, 7a, 7b, 8 | N/A | N/A | N/A |
| hsa-miR-429 | 6, 7a, 7b, 8 | N/A | N/A | N/A |
| hsa-miR-7-5p | 6, 7a, 7b, 8 | N/A | N/A | N/A |
| hsa-miR-9-5p | 6, 7a, 7b, 8 | N/A | N/A | N/A |
| hsa-miR-96-5p | 6, 7a, 7b, 8 | N/A | N/A | N/A |
| ^1^Associated with mRNAs with a raw p-value <0.05, not necessarily and FDR<0.05.  ^2^miRNAs in more than one seed category were not included in any statistical tests. | | | | |
